# Compassion from Others and Organisational Mental Health: Ethical Interpretations through Religious and Secular Frameworks

**DOI:** 10.1192/j.eurpsy.2026.10466

**Published:** 2026-07-31

**Authors:** C. Lazzari, P. Crawford, Y. Kotera

**Affiliations:** 1 Psychiatry, International Centre for Healthcare and Medical Education (ICHME), London; 2 Health Sciences, University of Nottingham, Nottingham, United Kingdom; 3 Center for Infectious Disease Education and Research, University of Osaka, Osaka, Japan; 4 Department of Social Sciences, Azerbaijan University, Azerbaijan, Azerbaijan

## Abstract

**Introduction:**

This study examines how working-age adults perceive compassion received in organizations, analyzing the impact of religious and secular belief systems. It fills a gap in organizational research by exploring how spirituality, culture, and personal values influence expectations and interpretations of empathetic behavior in professional relationships.

**Objectives:**

This study aims to explore how working-age adults understand and interpret compassion received from others in organizational settings, paying close attention to the role of religious and secular belief systems. It aims to identify the ethical foundations—whether spiritual, cultural, or experiential—that shape perceptions of received compassion in the workplace, and to analyze how these belief systems influence relational expectations and expressions of compassion in professional environments.

**Methods:**

A total of 285 adults, aged 18 to 65, living in the United States, participated in this research. Participants were recruited through anonymous online opportunistic sampling, which allowed for a broad demographic representation and diverse perspectives. Chi-square Goodness of Fit Index (GFI) statistics and Cohen’s *w* effect size were used to analyze the results.

**Results:**

When asked to identify the primary framework shaping their understanding of compassion in organizational settings, 34.39% of participants cited common sense, followed by religious beliefs (30.18%), personal experiences (24.56%), and company culture (10.18%). Other responses made up only 0.7%. This distribution was statistically significant (GFI: *p* < 0.001), with a large effect size (w = 1.63). The study also looked at how religion or faith influences compassionate behavior in the organisations. A majority (58.95%) agreed that religion or faith can influence compassionate actions and interpretations, while 28.77% disagreed, and 12.28% found such influence irrelevant. Chi-square analysis confirmed these differences as significant (GFI: *p* < 0.001), with a large effect size (*w* = 1.42).

**Conclusions:**

These findings underline the different interpretive frameworks people use when evaluating compassion from others in organizational settings. Common sense and secular ethics are the most common guiding principles, although religious influence remains important. The results suggest that both secular and spiritual belief systems play a key role in shaping expectations and behaviors related to compassion and moral responsibility at work. As organizations increasingly focus on emotional intelligence and interpersonal dynamics, understanding the range of interpretive frameworks may be essential for fostering inclusive and compassionate professional environments.

**Disclosure of Interest:**

None Declared

